# Neoadjuvant TACE combined with chemo-immunotherapy for giant triple-negative breast cancer: a case report

**DOI:** 10.3389/fimmu.2026.1739817

**Published:** 2026-02-18

**Authors:** Jie Tan, Zhuo Li, Zhi-Jun Li, Feng-Lin Kang, Hua Xiang, Peng Yan

**Affiliations:** 1 Department of Interventional Vascular Surgery, Hunan Provincial People’s Hospital (The First Affiliated Hospital of Hunan Normal University), Changsha, China; 2 Department of Interventional Vascular Surgery, Hunan Cancer Hospital, The Affiliated Cancer Hospital of Xiangya School of Medicine, Central South University, Changsha, Hunan, China

**Keywords:** case report, pathological complete response, systemic chemo-immunotherapy, transarterial chemoembolization, triple-negative breast cancer

## Abstract

Giant, ulcerated triple-negative breast cancer (TNBC) presents major therapeutic challenges. We report a 39-year-old woman with a 14.3 cm ulcerated TNBC (cT4bNxM0) who developed infection after intravenous chemotherapy. To achieve rapid local control, a multidisciplinary team elected to perform transarterial chemoembolization (TACE), followed by systemic chemo-immunotherapy using nab-paclitaxel and the PD-1 inhibitor Toripalimab. This multimodal approach led to marked tumor regression and improvement of skin ulceration, enabling curative surgery with modified radical mastectomy and complex reconstruction. Pathology postoperative showed a complete response (Miller-Payne grade 5, ypT0N0M0). This single case suggests that integrating TACE with multimodal neoadjuvant treatment sequences may be feasible in selected patients with extreme presentations of locally advanced TNBC, particularly in patients with extensive tumor burden or poor tolerance to systemic therapy. The approach may enhance local control and elicit synergistic immune activation, offering a potential paradigm for managing extreme TNBC presentations. Further studies are required to clarify the safety, efficacy, and potential immunomodulatory effects of this approach.

## Introduction

Giant, locally advanced triple-negative breast cancer (TNBC) represents a confluence of poor prognostic factors such as immense tumor volume, advanced local invasion, and an aggressive molecular subtype (1). Giant breast cancer, defined as tumors larger than 5 centimeters, is a rare cancer characterized by rapid growth that often outgrows the blood supply, leading to necrosis, skin ulcers, and infection. Treatment becomes even more challenging when the tumor is in the cT4b stage (tumor with skin ulceration) and has the TNBC phenotype, which accounts for 15-20% of breast cancers and is associated with early recurrence and poor prognosis (2).

Neoadjuvant therapy (NAT) is the standard of care for high-risk, non-metastatic TNBC (3). The goals are to downstage the tumor to facilitate surgery and to assess *in vivo* treatment sensitivity, with achievement of a pathological complete response (pCR) being a strong indicator of good long-term survival. The modern treatment backbone, established by the landmark KEYNOTE-522 trial, consists of platinum-taxane and anthracycline chemotherapy combined with the PD-1 inhibitor pembrolizumab (4).

However, this systemic approach faces significant obstacles in settings with extremely high tumor burden, such as the 14.3-cm tumor presented here. The sheer size, extensive necrosis, and high interstitial pressure can create a physiological barrier, impeding the effective delivery of systemic agents (5). Furthermore, pre-existing ulceration and infection increase the risk of sepsis during myelosuppressive chemotherapy. We hypothesized that incorporation of a potent locoregional treatment, transarterial chemoembolization (TACE), could rapidly debulk tumors, control local complications, and synergize with systemic chemoimmunotherapy, providing a “bridge” to radical treatment such as surgery.

## Case presentation

A 39-year-old female presented with a right breast mass that had been progressively enlarging for over a year following an initial diagnosis of invasive ductal carcinoma, for which she did not receive standard treatment due to personal financial issues. The tumor growth was associated with bloating, discomfort, and eventually, skin ulceration with fluid discharge.

A core needle biopsy performed one month before admission confirmed invasive ductal carcinoma, grade II. Immunohistochemistry (IHC) revealed a triple-negative profile: ER-negative, PR-negative, and HER2-negative (1+), with a Ki-67 proliferation index of 60%. The patient received one cycle of neoadjuvant chemotherapy with the TAC regimen (paclitaxel liposome, cyclophosphamide, and epirubicin). While the tumor softened, she developed a severe fever unresponsive to initial symptomatic treatment.

**Figure 1:**
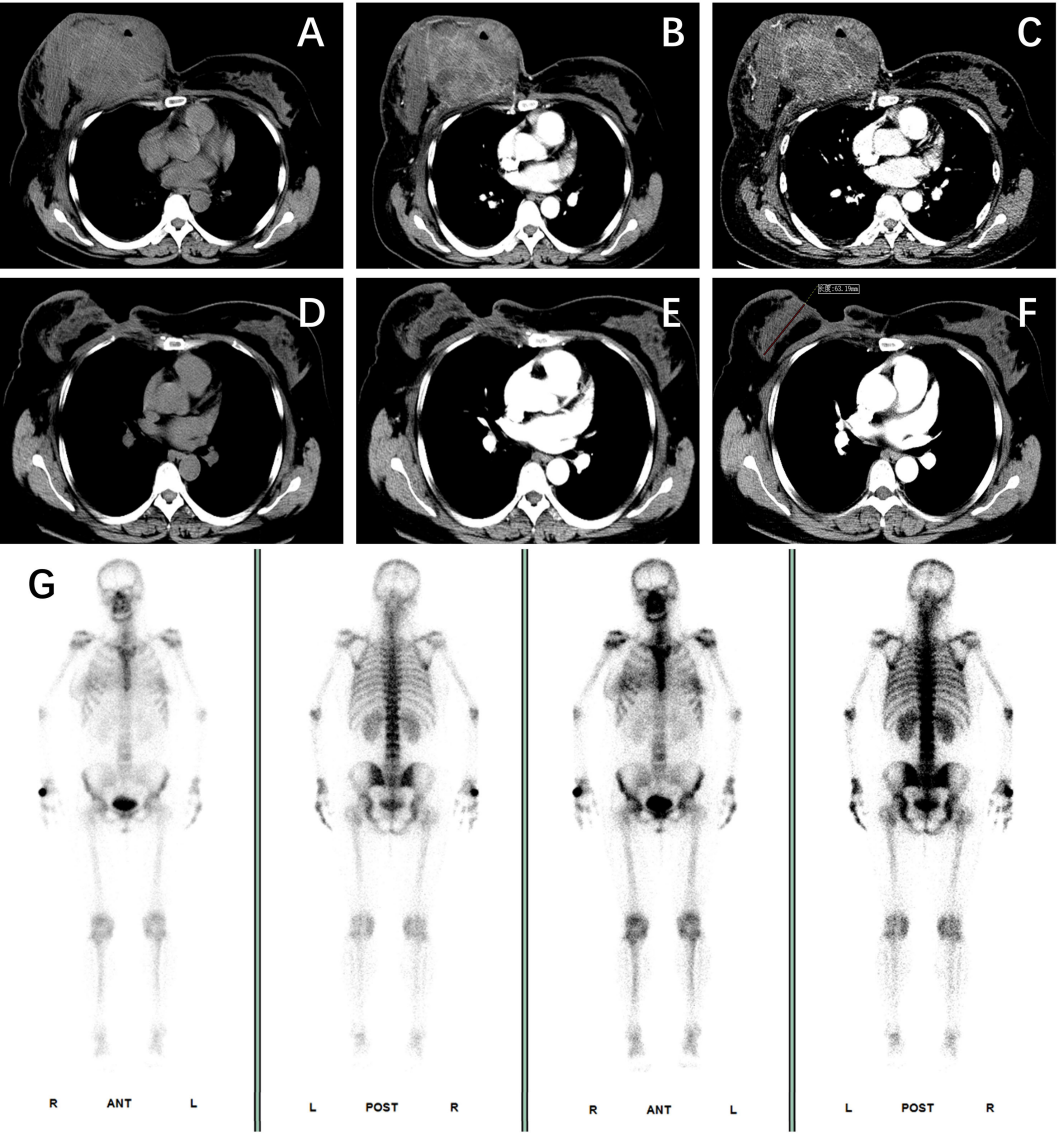
Radiographic response to neoadjuvant therapy. *Baseline (pre-treatment)*: **(A-C)** Contrast-enhanced axial CT images showing a large, heterogeneously enhancing mass in the right breast, with a maximum diameter of 14.3 cm and visible gas cavities; delayed enhanced CT showed persistent enhancement. *Post-neoadjuvant therapy*: **(D-F)** Follow-up CECT scans after treatment demonstrated a marked therapeutic response with significant regression of the breast tumor and axillary lymph nodes. **(G)** Anterior and posterior views from a whole-body bone scan showing no evidence of distant osseous metastases.

**Figure 2:**
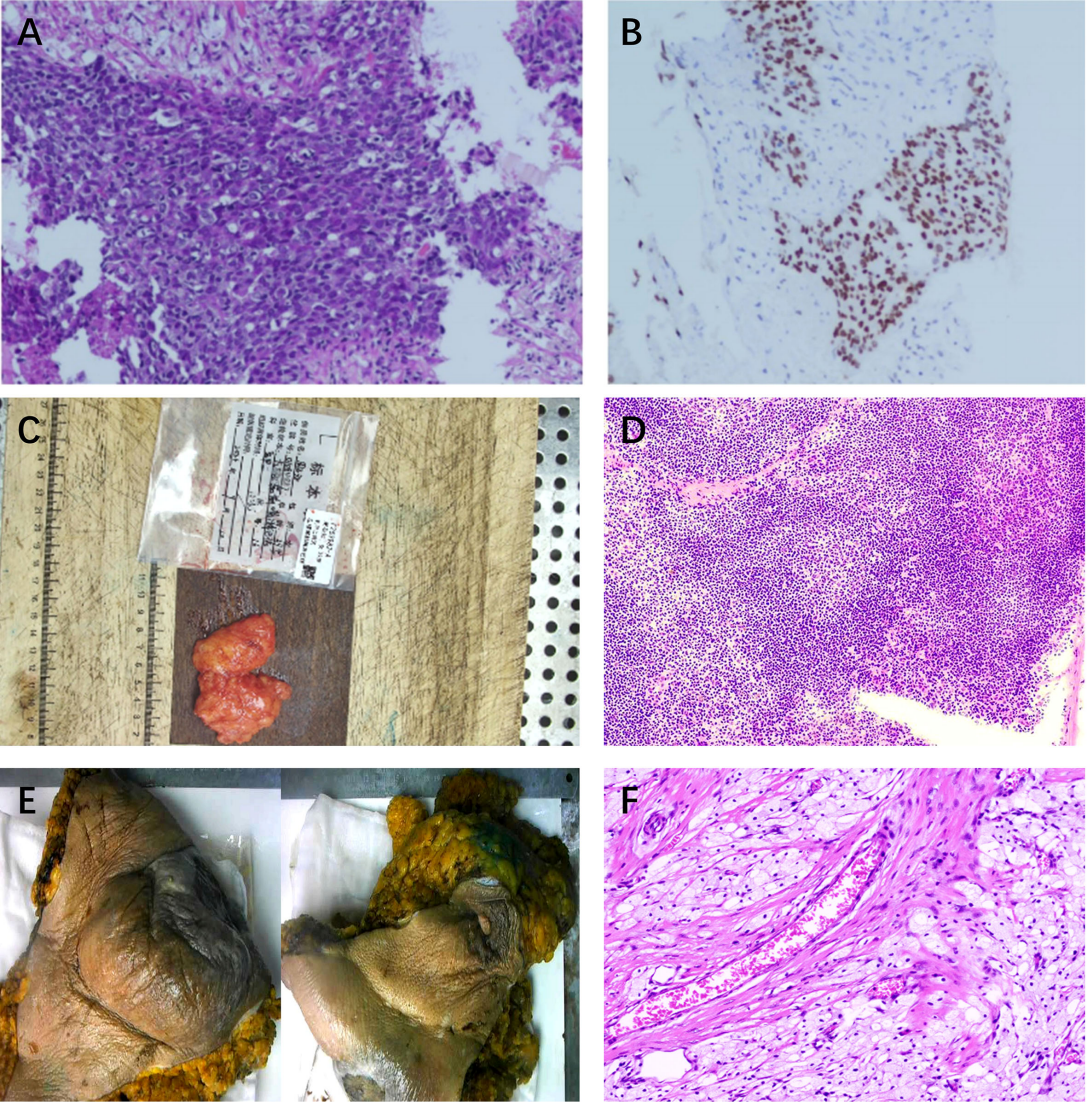
Histopathological assessment at baseline and after completion of neoadjuvant therapy. *Pre-treatment (baseline)*: **(A)** Diagnostic core biopsy showing Grade II invasive ductal carcinoma (H&E, ×200). **(B)** High Ki-67 proliferation index of ~60% (IHC). The tumor was triple-negative, basal-like (SOX-10+), with 6% PD-L1 expression (×200). *Post-neoadjuvant therapy*: **(C, D)** Intraoperative pathological assessment of sentinel lymph nodes showing **(C)** gross specimen and **(D)** H&E staining confirming reactive hyperplasia with no evidence of metastasis (0/14 nodes) (H&E, ×100). **(E-F)** Post-treatment mastectomy specimen showing **(E)** macroscopic appearance of the total mastectomy specimen and **(F)** postoperative pathology showed Miller-Payne Grade 5 with no residual tumor (H&E, ×200).

Upon admission, contrast-enhanced computed tomography (CECT) revealed a large, heterogeneously enhancing right breast mass measuring 14.3 cm at its longest diameter, with internal gas, indicative of necrosis and infection (**Figures 1A-C**). The ultrasound (US) presented with multiple enlarged hypoechoic nodules in the right axilla. The clinical stage was cT4bNxM0. After her fever resolved with broad-spectrum antibiotics, an expanded IHC panel showed the tumor was GATA3(-), SOX-10(+), and programmed death-ligand 1 (PD-L1) [SP263] positive (Combined Positive Score [CPS] = 6) (**Figures 2A, B**).

**Figure 3:**
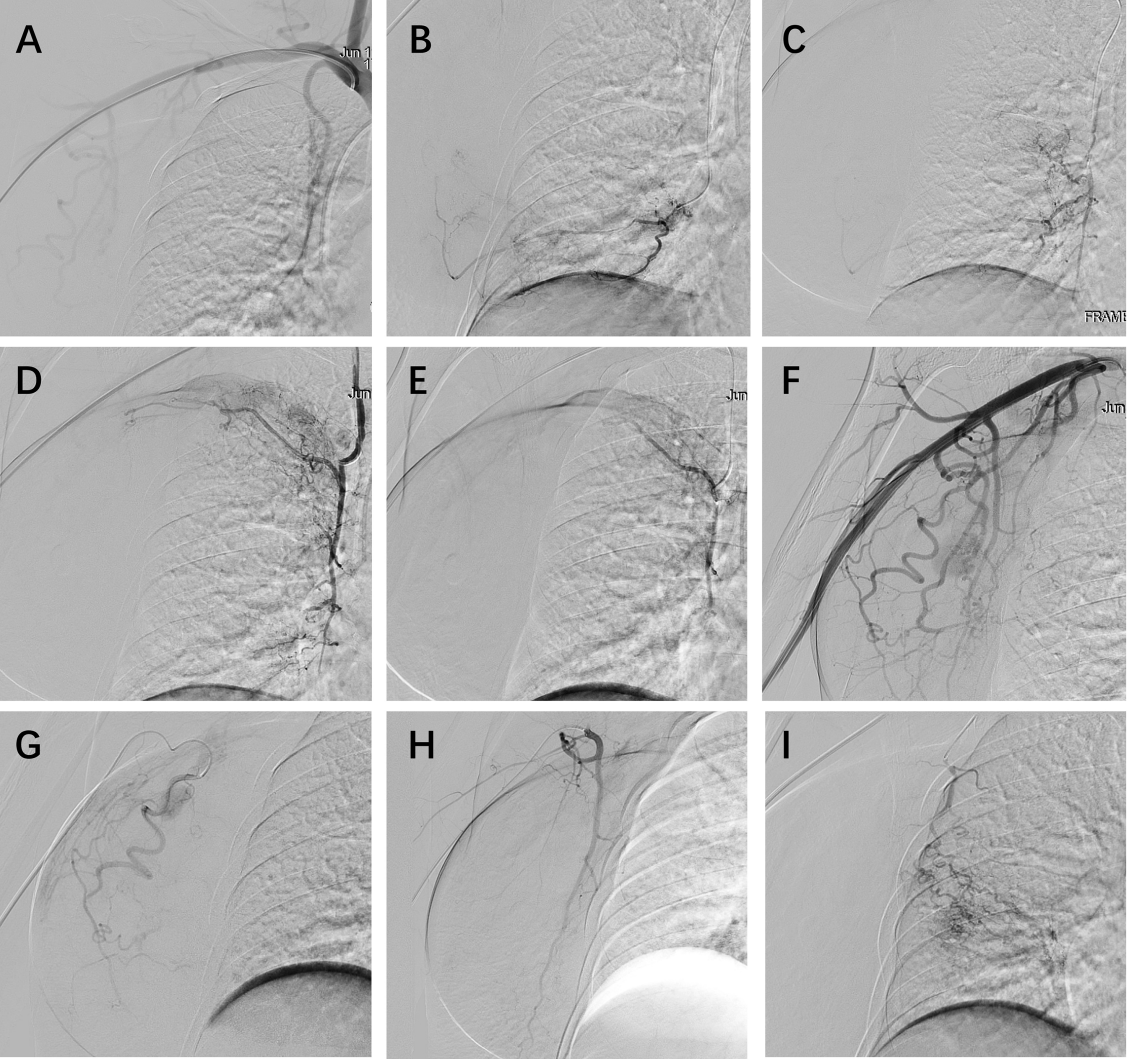
Transarterial chemoembolization (TACE) for right breast carcinoma. **(A)** Initial survey angiography of the right subclavian artery. **(B-D)** Superselective catheterization of branches from the right internal thoracic artery, revealing tumor supply, followed by embolization. **(E)** Post-embolization angiography confirms successful devascularization of the targeted internal thoracic artery branches. **(F)** Subsequent right axillary artery angiography demonstrates additional hypertrophied tumor-feeding vessels, including the thoracoacromial, lateral thoracic, and subscapular arteries, with extensive tumor and lymph node staining. **(G-I)** Superselective angiography and embolization were then performed on the right thoracoacromial artery **(G)**, lateral thoracic artery **(H)**, and subscapular artery **(I)** to achieve comprehensive tumor embolization. .

Given the immense tumor burden, rapid local progression, and concomitant infection, the case was discussed in a multidisciplinary team (MDT). Considering the high risk of further local deterioration and the limited likelihood of achieving timely tumor control with systemic therapy alone, TACE was selected as an upfront locoregional intervention to achieve rapid cytoreduction and local disease stabilization. Angiography identified feeding vessels from the internal and external thoracic arteries, which were super-selected (**Figure 3**). Carboplatin 300 mg was mixed with 100 ml of normal saline and slowly injected. Then, triacryloyl gelatin microspheres (TAGM) (700-900 um, Embosphere, Meriton) were used to embolize the tumor supply arteries. The patient experienced post-embolization pain, which was controlled after symptomatic treatment, and no other complications or adverse events occurred. Four days after TACE, once the patient’s general condition had stabilized, systemic neoadjuvant therapy was initiated. Given the non-standard clinical scenario and deviation from conventional KEYNOTE-522–based regimens, a revised chemotherapy–immunotherapy combination was selected. The patient received nab-paclitaxel (400 mg) combined with the PD-1 inhibitor Toripalimab (240 mg) every four weeks. At baseline before systemic therapy, laboratory tests demonstrated elevated inflammatory markers, which were considered to be associated with the post-embolization inflammatory response. Specifically, the white blood cell count was 11.97 × 10^9^/L, with a neutrophil count of 10.26 × 10^9^/L. Inflammatory biomarkers were also increased, including C-reactive protein (CRP) at 65.770 mg/L and procalcitonin at 0.251 ng/mL.

**Figure 4:**
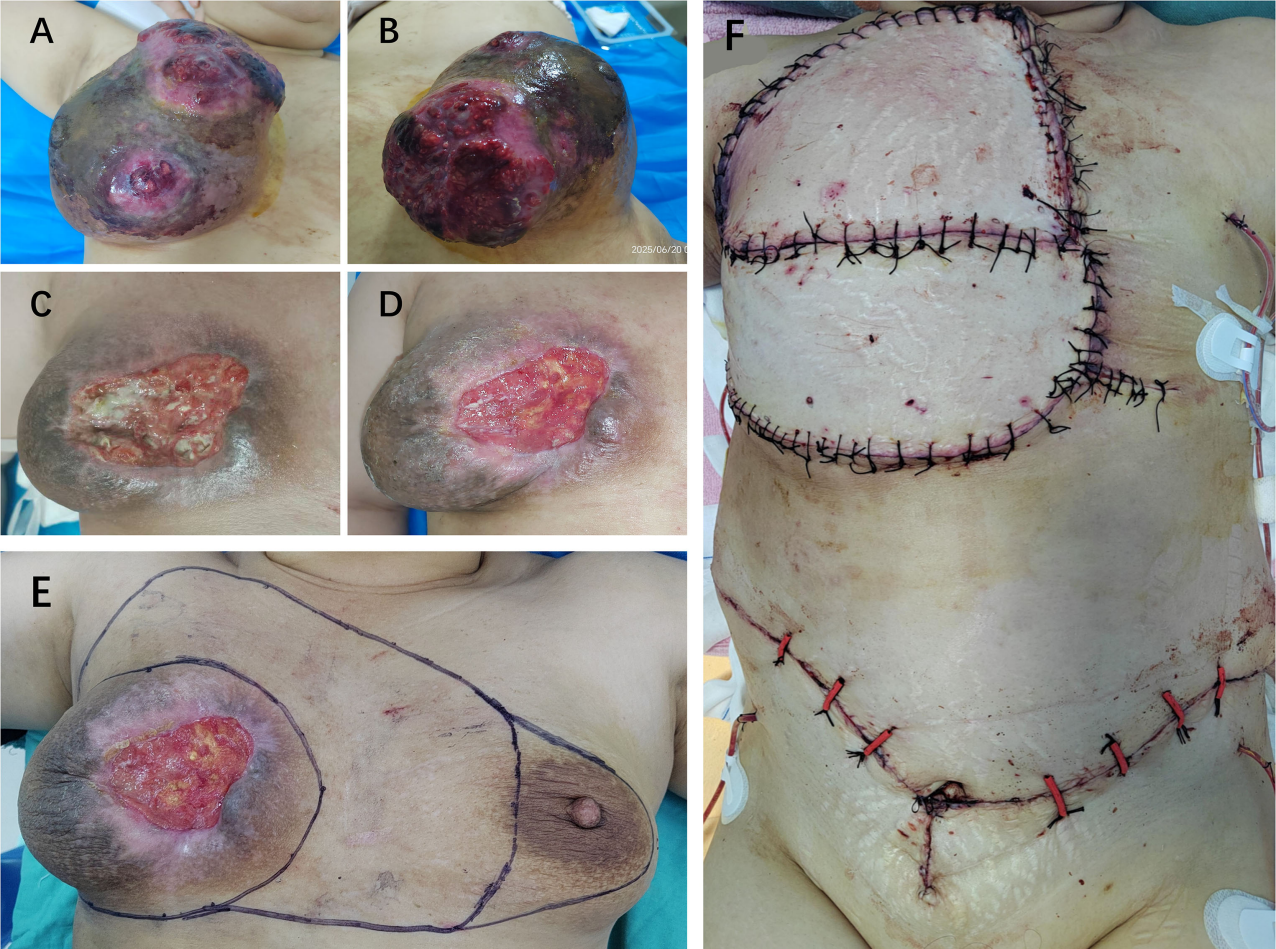
Clinical progression and surgical management of locally advanced breast cancer. **(A, B)** Baseline presentation on June 20, showing a giant, exophytic, and ulcerated right breast mass with significant necrosis. **(C)** Appearance of the tumor on August 14 after transarterial chemoembolization (TACE), four cycles of chemotherapy, and three cycles of immunotherapy, demonstrating a positive clinical response with reduced tumor size and improved ulceration. **(D)** Preoperative status on September 4, showing further consolidation of the therapeutic response. **(E)** Surgical planning for wide local excision. **(F)** Postoperative appearance of the chest wall after radical mastectomy, axillary lymph node dissection, and defect reconstruction with a skin flap.

**Figure 5:**
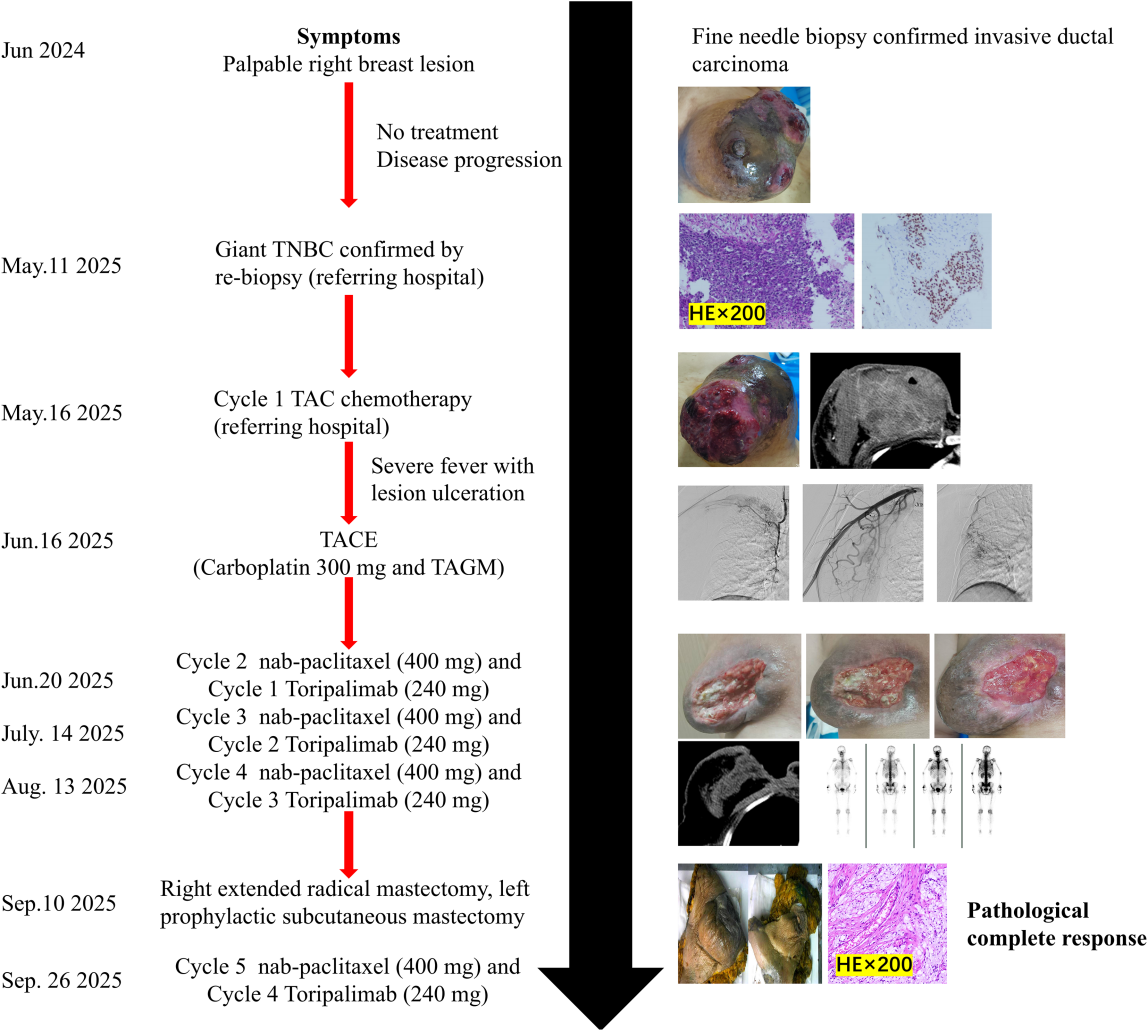
Timeline of diagnosis, treatments, and outcomes.

After four cycles of chemotherapy and three cycles of immunotherapy, she demonstrated a profound clinical response. The right breast mass shrank significantly, and the overlying skin ulcer healed completely. **Figure 4** presents the macroscopic changes of the right breast mass after preoperative neoadjuvant therapy. Repeat CECT confirmed marked tumor regression in the breast and axillary lymph nodes (**Figures 1D-F**), and a whole-body bone scan showed no evidence of distant metastases (**Figure 1G**). The patient subsequently underwent curative-intent surgery, which included a right extended radical mastectomy, left prophylactic subcutaneous mastectomy, and bilateral breast reconstruction with free deep inferior epigastric perforator (DIEP) flaps. Intraoperative rapid pathology showed reactive hyperplasia of the left axillary sentinel lymph node (0/14), and no cancer was found in the frozen section (**Figure 2C, D**). Final pathology of the resected right breast and 43 axillary lymph nodes revealed a pathological complete response (pCR) (**Figures 2E, F**). No residual invasive carcinoma was identified in the tumor bed (ypT0N0M0), which showed extensive treatment-related changes consistent with a Miller-Payne grade 5 response (6). The patient recovered well, resumed adjuvant chemoimmunotherapy two weeks after surgery, and was discharged uneventfully. **Figure 5** illustrates the chronological timeline of the patient’s diagnosis, therapeutic interventions, and clinical outcomes in accordance with the CARE guidelines. **Supplementary Table 1** demonstrates the detailed treatment timeline and content.

## Discussion

Achieving a pCR in a 14.3-cm ulcerated TNBC is an exceptional outcome that highlights the potential of an aggressive multimodal neoadjuvant treatment strategy. pCR is the most powerful predictor of long-term survival in TNBC, with a Miller-Payne grade 5 response associated with a 5-year overall survival approaching 95% (7). In this case, the favorable outcome was observed following a deliberate deviation from standard treatment paradigms, tailored to address the unique biological and clinical challenges posed by a giant, locally advanced tumor.

A key component of this multimodal approach was the integration of TACE. While TACE is an established therapy for hepatocellular carcinoma and breast cancer liver metastases, its incorporation into a neoadjuvant chemo-immunotherapy framework for giant, ulcerated, primary TNBC has rarely been reported (8, 9). In this case, TACE served two critical functions. First, it provided immediate, potent locoregional control by delivering a high concentration of carboplatin directly to the tumor’s vascular supply and inducing ischemia, thereby potentially overcoming drug delivery barriers inherent to a massive necrotic tumor burden (10). This intervention rapidly stabilized the patient’s deteriorating local condition, controlled the infection, and created a safer window for intensive systemic therapy.

Second, and perhaps more importantly, TACE may function as a potent immunological primer, creating an “*in-situ* vaccine”. Preclinical and translational studies in liver and lung cancers have demonstrated that embolization-induced ischemic tumor necrosis triggers immunogenic cell death (ICD), characterized by the release of damage-associated molecular patterns (DAMPs) such as high-mobility group box 1 (HMGB1), adenosine triphosphate (ATP), and calreticulin exposure (11–13). These signals promote dendritic cell maturation, tumor antigen presentation, and subsequent T-cell priming. Animal models further suggest that arterial embolization increases intratumoral CD8^+^ T-cell infiltration and reshapes the tumor microenvironment toward a pro-inflammatory, immune-responsive state (14, 15).

The pathological findings of “abundant foamy histiocytes” (macrophages) and “cholesterol crystals” are direct evidence of the immune system’s response to this event. These macrophages are actively engulfing the lipid-rich debris from dead cancer cells, processing the tumor antigens, and functioning as antigen-presenting cells. This initial innate response successfully escalates into a powerful, adaptive T-cell attack, evidenced by the “multinucleated giant cell reaction and granuloma formation”. Granulomas are highly organized structures of immune cells, including T-cells, that form in response to persistent antigens, signifying the transformation of the tumor microenvironment from immunologically “cold” to “hot” (16). Importantly, such TACE-induced immune activation may also upregulate inhibitory immune checkpoints, including PD-L1 expression. In this context, subsequent PD-1 blockade with Toripalimab may have enhanced and prolonged antitumor T-cell activity by releasing inhibitory constraints on an already primed immune response (17). However, given the close temporal sequence of therapies, the individual contributions of TACE and systemic chemoimmunotherapy cannot be fully disentangled.

The tumor’s aggressive biology, marked by a high Ki-67 index and SOX-10 positivity, paradoxically may have rendered it highly vulnerable to this intensive multimodal assault (18). SOX-10 expression is associated with a poor prognosis in TNBC, correlating with higher grade and metastatic potential (19). However, the same features that drive this aggressive behavior—hyperproliferation and genomic instability—may also render it susceptible to cytotoxic chemotherapy and generate a rich landscape of neoantigens for the immune system to target. This “high-risk, high-reward” profile suggests that while standard treatments may be insufficient, aggressive combination therapies can induce significant therapeutic benefits. Following significant tumor downstaging, definitive surgery remains crucial to ensure local control because imaging cannot reliably exclude minimal residual disease. The extensive resection necessitated complex reconstruction with bilateral DIEP flaps, which demonstrated a commitment not only to oncological safety but also to restoring the patient’s quality of life and body image.

From a safety perspective, the absence of severe adverse events in this case is encouraging but warrants cautious interpretation. Potential risks of TACE in breast cancer include non-target embolization, chest wall or skin ischemia, delayed wound healing, and inflammatory injury to surrounding tissues (9, 10). In addition, combining TACE with immune checkpoint inhibitors raises theoretical concerns regarding amplified inflammatory responses, immune-related adverse events, and postoperative complications (20). However, careful patient selection, super-selective catheterization of tumor-feeding vessels, and close multidisciplinary monitoring likely mitigated these risks in the present case (21). Importantly, no clinically significant liver dysfunction, immune-related toxicity, or surgical complications were observed, suggesting that this strategy can be feasible and tolerable when applied judiciously.

Previous reports of TACE in breast cancer have largely focused on its palliative application for liver metastases or cytoreduction in unresectable locally advanced disease, most commonly in combination with chemotherapy alone (22, 23). In contrast, the present case explores a distinct clinical paradigm in which TACE was intentionally integrated into a neoadjuvant chemo-immunotherapy sequence for a giant, ulcerated, primary TNBC. This upstream use of TACE, followed closely by PD-1–based systemic therapy, represents a strategic shift from symptom control toward immune modulation and curative intent, thereby highlighting a potential new avenue for multimodal treatment design in highly selected patients.

The primary limitation of this report is that it is a single case, and its success cannot be generalized. Furthermore, due to the close temporal proximity between TACE and systemic therapy, definitive attribution of efficacy to any single modality is not possible. Nevertheless, as a proof-of-concept, it strongly suggests that integrating TACE into a neoadjuvant chemoimmunotherapy platform is a feasible and potentially transformative strategy for carefully selected patients with giant, complex TNBC. Another limitation of this report is the relatively short follow-up period, which precludes assessment of long-term recurrence and survival outcomes. Ongoing follow-up is planned to monitor the durability of the response and late adverse events. Finally, frozen tumor samples and comprehensive peripheral immune panel data were not available in this case, precluding retrospective assessment of these parameters. Future studies should therefore incorporate predefined immune baseline assessments both before and after locoregional therapy to better delineate the immunomodulatory effects of sequential embolization and immune checkpoint blockade.

## Conclusion

This case report details the successful management of an exceptionally challenging giant, ulcerated TNBC. By integrating locoregional TACE with systemic chemo-immunotherapy in the neoadjuvant setting, we achieved a pCR, enabling curative-intent surgery. This innovative, multimodal strategy demonstrates that for patients with extreme tumor burdens, a tailored approach combining rapid local debulking with state-of-the-art systemic therapy can overcome the limitations of standard regimens and produce profound therapeutic responses. The potential synergy between TACE-induced *in-situ* vaccination and immune checkpoint inhibition offers a promising avenue for future research in this high-risk patient population.

## Data Availability

The original contributions presented in the study are included in the article/**Supplementary Material**. Further inquiries can be directed to the corresponding authors.
