# Supplementary material for: Neoadjuvant TACE combined with chemo-immunotherapy for giant triple-negative breast cancer: a case report
Source: Front Immunol. 2026 Feb 18;17:1739817. doi: 10.3389/fimmu.2026.1739817 (PMC12956800; doi:10.3389/fimmu.2026.1739817)
Supplement: Supplementary file 1 [file Table1.docx]

Supplementary Table 1. Detailed Treatment Timeline and Multimodal Therapeutic Interventions

| **Treatment Phase** | **Date / Timeframe** | **Regimen / Procedure** | **Dose & Schedule** | **Number of Cycles / Sessions** | **Rationale / Notes** |
| --- | --- | --- | --- | --- | --- |
| Initial systemic therapy (Referring hospital) | Prior to referral | TAC chemotherapy | Paclitaxel liposome 230 mg; Epirubicin 120 mg; Cyclophosphamide 0.8 g; administered intravenously | 1 cycle | Initiated at a referring hospital for presumed locally advanced TNBC. Treatment was discontinued due to worsening ulceration, infection, and inadequate local control. |
| Locoregional therapy | Day 0 | Transarterial chemoembolization (TACE) | Carboplatin 300 mg diluted in 100 mL normal saline, slowly infused intra-arterially; followed by embolization with triacryloyl gelatin microspheres (700–900 μm, Embosphere, Merit Medical) | 1 session | Multidisciplinary decision due to massive tumor burden, active infection, and poor suitability for immediate systemic therapy. Aimed at rapid locoregional control and cytoreduction. |
|  |  | TACE technical details | Super-selective catheterization of tumor-feeding branches from the internal thoracic artery, thoracoacromial artery, lateral thoracic artery, and subscapular artery | — | Post-embolization angiography confirmed effective devascularization. Only transient post-embolization pain occurred, with no major complications. |
| Systemic neoadjuvant therapy (revised regimen) | 4 days after TACE | Nab-paclitaxel + Toripalimab | Nab-paclitaxel 400 mg IV every 4 weeks; Toripalimab 240 mg IV every 4 weeks | Nab-paclitaxel: 4 cycles; Toripalimab: 3 cycles | Selected as a modified neoadjuvant chemo-immunotherapy strategy considering infection risk, poor local condition, and potential immunomodulatory synergy following TACE. |
| Response assessment | During neoadjuvant therapy | Clinical and radiologic evaluation | Contrast-enhanced CT, whole-body bone scan, and clinical examination | — | Demonstrated marked tumor regression, resolution of ulceration, no distant metastasis, and improvement in local inflammatory status. |
| Radical surgery | After completion of neoadjuvant therapy | Radical mastectomy + axillary lymph node dissection + reconstruction | Total mastectomy; axillary lymph node dissection; bilateral DIEP flap reconstruction | 1 surgery | Performed after significant tumor downstaging to achieve definitive local control and address extensive chest wall defect. |
| Pathological outcome | Postoperative | Histopathological assessment | — | — | Pathological complete response (pCR), Miller–Payne Grade 5; no residual invasive carcinoma; lymph nodes negative (0/14). |
| Adjuvant therapy & follow-up | Ongoing | Adjuvant systemic therapy and surveillance | According to institutional protocol | — | Patient recently completed surgery and adjuvant therapy; follow-up ongoing with no evidence of recurrence at last assessment. |
